# Intermediate-onset colorectal cancer: A clinical and familial boundary between both early and late-onset colorectal cancer

**DOI:** 10.1371/journal.pone.0216472

**Published:** 2019-05-16

**Authors:** María Arriba, Carmen Sánchez, Alfredo Vivas, OA Nutu, Daniel Rueda, Sandra Tapial, Yolanda Rodríguez, Lorena Brandáriz, Juan L. García, Damián García-Olmo, Ajay Goel, Rogelio González-Sarmiento, Miguel Urioste, José Perea

**Affiliations:** 1 Department of Clinical Biochemistry, “Gregorio Marañón” University Hospital, Madrid, Spain; 2 Department of Surgery, “12 de Octubre” University Hospital, Madrid, Spain; 3 Molecular Biology Laboratory, “12 de Octubre” University Hospital, Madrid, Spain; 4 Centre for Biomedical Research of “12 de Octubre” University Hospital, Madrid, Spain; 5 Department of Pathology, “12 de Octubre” University Hospital, Madrid, Spain; 6 Department of Surgery, “Fundación Jiménez Díaz” University Hospital, Madrid, Spain; 7 Health Research Institute, “Fundación Jiménez Díaz” University Hospital, Madrid, Spain; 8 Molecular Medicine Unit, Department of Medicine, Institute of Molecular and Cellular Biology of Cancer (IBMCC) and Biomedical Research Institute of Salamanca (IBSAL), University of Salamanca-SACYL-CSIC, Salamanca, Spain; 9 Center for Gastrointestinal Research, Center for Translational Genomics and Oncology, Baylor Scott & White Research Institute, Charles A, Sammons Cancer Center, Baylor University Medical Center, Dallas, Texas, United States of America; 10 Centre for Biomedical Network Research on Rare Diseases (CIBERER), Institute of Health Carlos III, Madrid, Spain; 11 Familial Cancer Clinical Unit, Spanish National Cancer Centre (CNIO), Madrid, Spain; Howard University, UNITED STATES

## Abstract

Comparative studies of colorectal cancer (CRC) according to the age of onset have found differences between early-onset CRC (EOCRC) and late-onset CRC (LOCRC). Using this as a starting point, we wished to determine whether intermediate-onset CRC (IOCRC) might also be considered as an independent group within CRC. We performed a retrospective comparative study of the clinicopathological and familial features, as well as of the symptoms and their duration, of a total of 272 subjects diagnosed with CRC classified into three groups according to the age-of-onset (98 EOCRC, 83 IOCRC and 91 LOCRC). The results show that from a clinicopathological point of view, IOCRC shared certain features with EOCRC (gender, prognosis), and with LOCRC (multiple primary CRCs), whereas it also had characteristics that were specific for IOCRC (mean number of associated polyps). A gradual progression was observed from EOCRC to LOCRC from a greater family aggregation to sporadic cases, in parallel with a change of Lynch Syndrome cases to the sporadic microsatellite instability pathway, with the IOCRC being a boundary group that is more related to EOCRC. With respect to symptoms, duration and correlation with stages, IOCRC appeared more similar to EOCRC. Clinically, IOCRC behaves as a transitional group between EOCRC and LOCRC, with features in common with both groups, but also with IOCRC-specific features. Excluding cases with familial cancer history, the awareness for EOCRC diagnosis should be extended to IOCRC.

## Introduction

Colorectal cancer (CRC) represents the third most common malignancy in developed countries [1,2]. Its pathogenesis is tightly related to the loss of genomic stability involving one of at least three major molecular pathways: chromosomal instability (CIN), microsatellite instability (MSI), and CpG island methylator phenotype (CIMP). The CIN pathway (also called the “suppressor” pathway) accounts for the majority of spontaneous CRCs (85%) [3], and the MSI pathway (also called the “mutator” pathway) accounts for about 15% of CRCs; these latter cases are mainly related to Lynch syndrome (LS) and epigenetic silencing of the *MLH1* gene [4,5]. On the other hand, the CIMP pathway, by which methylation in the CpG islands causes the silencing of genes, involved in almost 40% of CRCs [6,7].

Since the risk of developing CRC increases as individuals get older, early-onset CRC (EOCRC) represents a rare entity commonly related to hereditary forms of the disease (2–8% of all CRCs) [8]. It has been observed that MSI does not explain the majority of EOCRC cases, and some authors have suggested that EOCRC should not be considered to be intimately associated with hereditary forms of CRC [8–11]. From a clinical point of view, sporadic early-onset tumors are more aggressive and confer poorer survival than the late-onset ones. They are more frequently associated with invasive phenotypes, early metastasis, and familial clustering [8,9,12]. From a molecular point of view, EOCRCs show substantial dissimilarities regarding the CIN pattern in comparison with LOCRC [13] and seem to be more frequently to LINE-1 hypomethylation [14]. Consequently, it has been proposed that the molecular basis of CRC might be different for different ages of onset [13].

Taking this as a starting point, the aim of the present study was to investigate whether intermediate-onset CRC (IOCRC) (51–69 years old at the time of diagnosis) might also be considered as a different group within CRC according to the clinicopathological features of these tumors or if, on the contrary, it should be interpreted as a transitional group between EOCRC and LOCRC in which the clinicopathological features progressively vary from those typical in EOCRC to those typical in LOCRC.

## Materials and methods

### Patients, samples and data collection

We compared three groups of patients diagnosed with CRC differing in the age at onset. We collected 98 individuals diagnosed at an age of 50 years or younger (Early-onset CRC: EOCRC), 83 individuals diagnosed at an age of 51–69 years (Intermediate-onset CRC: IOCRC), and 91 individuals diagnosed at an age of 70 years or older (Late-onset CRC: LOCRC). All patients were selected during the same period at the Hospital Universitario 12 de Octubre in Madrid and provided written consent. In case of death of the index case, a first-degree relative provided the consent. The study was approved by the Ethics Committee of the “12 de Octubre” University Hospital (Madrid).

Family history of cancer (including at least three generations) and clinicopathological information was obtained for each patient with a follow-up of at least 5 years from surgery. Personal and clinicopathological information included age of onset, gender, location of the CRC, grade of tumor differentiation, mucin production, the presence of “signet ring” cells, stage at diagnosis, the existence and type of polyps during follow-up and the presence of synchronous or metachronous CRCs (SCRC or MCRC) were collected.

### Symptoms and their duration at the time of diagnosis

All patients reported their symptoms and the duration of these symptoms at their first visit. For incidental findings resulting in a diagnosis of CRC (e.g. anemia, screening strategies…), the duration of the symptoms was considered as zero. Symptoms were defined as intestinal bleeding (hematochezia, melena), changes in bowel habit (constipation and/or diarrhea), constitutional syndrome (weight loss, anorexia and/or asthenia), anorectal symptoms (pain, tenesmus), abdominal-related (pain, mass), unspecific symptoms (fatigue, bloating, nausea, anemia-related symptoms), emergency diagnosis (bowel obstruction, acute abdominal pain) and incidental diagnosis, as mentioned before. Finally, bleeding was defined as self-limited (when colon bleeding was subsequent to therapeutic interventions), as associated with other symptoms, or as isolated.

### Molecular characterization of the tumors

A pathologist performed microscopic inspection of the tumor tissue of paraffin-embedded samples from the index cases, and samples with more than 70% of tumor cells in the neoplastic material were considered adequate for further analysis. The protocol for DNA isolation was as previously reported [13].

We used the Bethesda panel to assess the MSI status and considered two or more altered markers as a positive result [15]. We also considered tumors showing by immunohistochemistry lack of expression of at least one mismatch repair (MMR) protein as MSI tumors. All MSI cases were analyzed for the *BRAF* V600E mutation and hypermethylation of the *MLH1* gene promoter in order to confirm their sporadic nature [9], and prescreened for germline mutations in *MLH1*, *MSH2* and *MSH6* by denaturing gradient gel electrophoresis (DGGE), denaturing high-performance liquid chromatography (dHPLC), or high-resolution melting (HRM) analysis. Primers and denaturing/melting conditions were as previously reported, with slight modifications [16]. When an anomalous band or pattern was observed by DGGE, dHPLC or HRM, the PCR product was sequenced using the BigDye Terminator v3.1 Cycle Sequencing Kit (Applied Biosystems) and analyzed with an ABI Prism 3130 Genetic Analyzer (Applied Biosystems).

We also tested *KRAS* mutational status for known activating mutations by using polymerase chain reaction amplification of codons 12,13, and 61, as well as by targeted next-generation sequencing of all coding regions.

### Statistical analyses

Continuous variables were expressed as mean values plus/minus standard deviation (SD), and categorical variables were expressed as number of cases and their percentage. For the assessment of associations between the age of onset and discrete variables, Pearson’s Chi Square (χ^2^) test was used. For comparisons of continuous variables in more than two groups, the Kruskal-Wallis test (nonparametric distributions) was used. Comparison of continuous variables was done using Student´s t-test. Statistical analysis was performed using SPSS 17.0 (SPSS Inc., Chicago, IL, USA), and differences were considered statistically significant when the p-value was <0.05.

## Results

### Clinical-pathological and familial features

Comparative results between the three CRC-onset categories are shown in Table 1.

**Table 1:** Clinical, pathological and familial features of the patients included in this study.

	EOCRC	IOCRC	LOCRC	p-value^a^
No. of patients	**98 (100)**	**83 (100)**	**91 (100)**	-
Mean age at onset **±** SD (years)	**41.01 ± 5.57**	**59.40 ± 4.87**	**77.49 ± 5.53**	-
Gender: Male Female	**63 (64)** **35 (36)**	**55 (66)** **28 (34)**	**43 (47)** **48 (53)**	0.017
Location: Right colon Left colon Rectum	**22 (22)** **44 (45)** **32 (33)**	**29 (35)** **25 (30)** **29 (35)**	**34 (37)** **24 (27)** **33 (36)**	**NS (0.052)**
Tumor differentiation^b^: Poor	**11/75 (15)**	**7/60 (12)**	**5/78 (6)**	**NS**
Mucin production^2^ “Signet ring” cells^2^	**19/75 (25)** **5/75 (7)**	**7/60 (12)** **0/60 (0)**	**18/78 (23)** **1/78 (1)**	**NS** 0.039
Astler-Coller Stage: A B C D	**25 (26)** **33 (34)** **18 (18)** **22 (22)**	**26 (31)** **20 (24)** **17 (21)** **20 (24)**	**6 (7)** **43 (47)** **18 (20)** **24 (26)**	0.002
Polyps during follow-up Average No. of polyps	**65 (66)** **5.68 [3.00]**	**63 (76)** **8.16 [5.00]**	**68 (75)** **5.09 [3.00]**	**NS** 0.027^c^
Type: Adenomatous Hyperplastic Mixed	**28 (43)** **7 (11)** **30 (46)**	**20 (32)** **3 (5)** **40 (63)**	**43 (63)** **4 (6)** **21 (31)**	0.002
Synchronous/Metachronous CRC	**8 (8)**	**25 (30)**	**22 (24)**	0.001
OS **±** SD (months) DFS **±** SD (months)	**75.77 ± 47.82** **66.00 ± 50.38**	**75.01 ± 41.43** **65.19 ± 46.95**	**39.81 ± 27.27** **31.49 ± 29.85**	<0.001^d^ <0.001^c^
Family history of cancer: Amsterdam II families Aggregation for LR neoplasms Sporadic cases	**18 (18)** **38 (39)** **42 (43)**	**5 (6)** **31 (37)** **47 (57)**	**1 (1)** **19 (21)** **71 (78)**	<0.001

Data shown in parenthesis represent percentages. Data shown in brackets represent median values.

^a^Statistical comparison was performed using Pearson’s Chi-Square (χ^2^) test.

^b^Percentages shown are based on varying total numbers as some cases were excluded because only one biopsy was taken (stage D), or because tumors were severely dysplastic with “in situ” carcinoma and it was not possible to study any other characteristic.

^c^Statistical comparison was performed using the Kruskal-Wallis test.

^d^Statistical comparison was performed using analysis of variance (ANOVA). CRC: Colorectal cancer. DFS: Disease-free survival. EOCRC: Early-onset colorectal cancer. IOCRC: Intermediate-onset colorectal cancer. LOCRC: Late-onset colorectal cancer. LR: Lynch-related. No.: Number. NS: Not significant. OS: Overall survival. SD: Standard deviation.

We observed a majority of male members in the younger groups (64% and 66% in EOCRC and IOCRC respectively; p = 0.017), whereas the male/female ratio was close to one in the LOCRC group. Differences regarding the tumor location according to the age at onset were near-significant, and it is noteworthy that left-sided CRCs were predominant in EOCRC. A mucinous histology and “signet ring” cells were also more frequent in the EOCRC group, but statistical significance was only reached in the latter case (p = 0.039; Table 1). Interestingly, the appearance of polyps during follow-up was more frequent in IOCRC and LOCRC (76% and 75%, respectively) than in EOCRC (66%) and the average number of polyps was significantly higher in the IOCRC group (p = 0.027), while polyps of the adenomatous type were less frequent in this group. Multiple primary CRCs (both S- and MCRC) were more frequent in IOCRC and LOCRC (30% and 24%, respectively, versus 8% in EOCRC). Regarding survival, EOCRC and IOCRC showed similar profiles (Overall survival: 75.77 and 75.01 months, respectively; Disease-Free survival: 66 and 65.19 months, respectively), while within the LOCRC group it was clearly worse (Overall survival: 39.81 months; and Disease-Free survival: 31.49 months). Finally, as expected, Amsterdam II criteria most frequently fulfilled in the EOCRC group (18% of cases), and another 39% of cases in this group showed an aggregation for LS neoplasms. This familial component decreased progressively through IOCRC (43% for both together) until reaching 22% in LOCRC.

### Symptoms and their duration at diagnosis

#### Global description

Information regarding the type of symptoms and their duration could be obtained from 231 and 220 patients, respectively (Table 2).

**Table 2:** Global shymptoms and their duration at the time of diagnosis.

	EOCRC	IOCRC	LOCRC	p-value^a^
No. of patients	**98 (100)**	**83 (100)**	**91 (100)**	-
Intestinal bleeding: Associated Isolated Self-limited	**38 (44)** **20 (52)** **9 (24)** **9 (24)**	**23 (35)** **14 (61)** **5 (22)** **4 (17)**	**25 (31)** **11 (44)** **2 (8)** **12 (48)**	**NS**
Abdominal symptoms	**24 (28)**	**19 (29)**	**8 (10)**	0.005
Constitutional syndrome	**21 (24)**	**15 (23)**	**16 (20)**	**NS**
Anorectal (excluding bleeding)	**1 (1)**	**2 (3)**	**1 (1)**	**NS**
Unspecific symptoms	**10 (12)**	**6 (9)**	**7 (9)**	**NS**
Incidental diagnosis: Anemia Screening Others	**16 (19)** **5 (31)** **7 (44)** **4 (25)**	**16 (25)** **3 (19)** **11 (69)** **2 (12)**	**26 (33)** **16 (62)** **7 (27)** **3 (11)**	0.017
Changes in bowel habit	**22 (26)**	**19 (29)**	**14 (18)**	**NS**
Emergency diagnosis	**6 (7)**	**3 (5)**	**8 (10)**	**NS**
Duration of symptoms (months)	**5.59 ± 8.59**	**4.46 ± 6.38**	**2.16 ± 3.42**	0.001^b^

Data shown in parenthesis represent percentages.

^a^Statistical comparison was performed using Pearson’s Chi Square test (χ^2^).

^b^Statistical comparison was performed using the Kruskal-Wallis test. EOCRC: Early-onset colorectal cancer. IOCRC: Intermediate-onset colorectal cancer. LOCRC: Late-onset colorectal cancer. No.: Number. NS: Not significant.

Considering all patients, intestinal bleeding was the most common symptom (37%), followed by changes in bowel habit (24%) and abdominal symptoms (22%). About 25% of patients were diagnosed incidentally, and 43% of these were detected due to screening strategies (as a result of familial and/or individual cancer history). Only 7% of the patients presented an emergency diagnosis and required emergency surgery.

We observed different clinical manifestations depending on the tumor location (S1 Table): right-sided tumors were more frequently associated with abdominal features (p = 0.005) and rectal tumors were more frequently associated with intestinal bleeding (p = 0.0001) and with changes in bowel habit (p = 0.03). On the other hand, left-sided tumors were most frequently related to bowel obstruction although statistical significance was not reached. As expected, anorectal symptoms were exclusively observed in rectal tumors. Finally, we also observed some clear issues regarding the correlation between type/duration of the symptoms and the stage of the cancer at the time of diagnosis (S2 Table). Thus, later stages of the disease were often associated with abdominal symptoms or constitutional syndrome (p = 0.005 and p = 0.006, respectively) whereas incidental diagnosis was more common in early stages. Accordingly, stage I of the disease showed the lowest duration of the clinical symptoms, and the increase correlated with increasing locoregional stage (stages I-III). Duration for remote Stage IV cases was slightly less than Stage II cases.

#### Comparative analysis of symptoms and duration depending on the age of onset

In our series, the duration of the symptoms was significantly longer in EOCRC (p = 0.001; Table 2), with intestinal bleeding being the most frequent symptom (44%; Table 2). Abdominal symptoms were common in both EOCRC and IOCRC (about 30% in both cases), but relatively uncommon in LOCRC (10%; p = 0.005) (Table 2). The LOCRC group showed the shortest average time of symptomatic disease. Incidental diagnosis was more frequent in this group, mostly due to anemia in analytical findings (p = 0.017; Table 2).

We also investigated the relationship between stages at diagnosis and clinical symptoms for each age group and observed that some features became different compared with those observed in the overall analysis (Table 3; S2 Table).

**Table 3:** Relationship between tumor staging and clinical symptoms for each age group.

	EOCRC				p-value^a^	IOCRC				p-value^a^	LOCRC				p-value^a^
	I	II	III	IV		I	II	III	IV		I	II	III	IV	
**No. of patients**	29	29	18	22	-	30	16	17	20	-	14	36	20	21	-
**Intestinal bleeding**: **Associated** **Isolated** **Self-limited**	7 2 0 5	13 8 3 2	10 6 3 1	8 4 3 1	NS	4 1 1 2	6 5 - 1	8 4 3 1	5 4 1 -	NS	3 - 1 2	9 4 1 4	5 3 - 2	8 4 - 4	NS
**Abdominal symptoms**	1	10	5	8	NS	2	5	3	9	NS	1	-	3	4	**0.045**
**Constitutional syndrome**	1	10	3	7	NS	-	4	3	8	**0.023**	2	4	5	5	NS
**Anorectal (excluding bleeding)**	-	1	-	-	NS	-	1	1	-	NS	-	1	-	-	NS
**Unspecific symptoms**	-	2	3	5	NS	1	2	-	3	NS	1	2	2	2	NS
**Incidental diagnosis**: **Anemia** **Screening** **Others**	12 3 6 3	3 2 1 -	- - - -	1 - - 1	**<0.001**	10 - 10 -	2 1 - 1	3 2 1 -	1 - - 1	**<0.001**	6 2 2 2	14 11 3 -	2 2 - -	4 1 2 1	NS
**Changes in bowel habit**	2	9	6	5	NS	-	9	4	6	**0.006**	1	5	5	3	NS
**Duration of symptoms (months)**	1.74 ± 5.60	7.00 ± 10.03	8.94 ± 11.58	4.71 ± 4.09	**<0.001**^b^	0.93 ± 1.91	4.60 ± 4.31	8.20 ± 10.21	4.17 ± 4.59	**0.003**^b^	1.64 ± 2.80	1.45 ± 2.79	3.57 ± 4.78	2.61 ± 3.40	NS^b^

Data shown in parenthesis represent percentages.

^a^Statistical comparison was performed using Pearson’s Chi Square test (χ2).

^b^Statistical comparison was performed using the Kruskal-Wallis test. No.: Number. NS: Not significant.

Thus, abdominal symptoms were prevalent in stage IV when all patients were considered, but not when each group was studied separately. The most differential group was IOCRC, with changes in bowel habit and constitutional syndrome being relatively frequent in stages II and IV, respectively (Table 3). Incidental diagnosis predominated in both EOCRC and IOCRC mainly due to screening strategies for stage I of the disease. Lastly, the duration of symptoms increased in EOCRC and IOCRC in parallel with the stage of the disease, except for stage IV where duration decreased with respect to foregoing stages (Table 3).

### Molecular analysis

In our series, a total of 27 tumors showed MSI (14 EOCRC cases, 4 IOCRC cases and 9 LOCRC cases) (Table 4) with a complete correlation with the immunohistochemical results. There were no differences between groups according to the MSI (14%, 6% and 10%, respectively).

**Table 4:** Comparison and description of the molecular features according to the age of onset.

	Total	EOCRC	IOCRC	LOCRC
**No. of patients**	272	98	83	91
**MSI**^a^**(%)**	27/256 (11)	14/98 (14)	4/67 (6)	9/91 (10)
**MMR genes affected**: *MLH1* *MSH2* *MSH6*	14 9 11	4 4 2	0 2 1	0 1 0
***BRAF* V600E mutation and/or *MLH1* promoter hypermethylation**	11	2	1	8
***KRAS* mutations**^a^	116/256 (45)	38/98 (39)	32/67 (48)	51/91 (56)

^a^ Percentages shown are based on varying total numbers as some cases were excluded because it was not possible to study all characteristics of all patients. EOCRC: Early-onset colorectal cancer. IOCRC: Intermediate-onset colorectal cancer. LOCRC: Late-onset colorectal cancer. MMR: Mismatch repair. MSI: Microsatellite instability. No.: Number.

Blood samples were taken from the MSI index cases to assess germline mutations in *MLH1*, *MSH2*, *MSH6* and *PMS2*. MSI tumors were also analyzed for the *BRAF* V600E mutation and *MLH1* promoter hypermethylation, in order to identify sporadic cases.

Of the 14 MSI-EOCRC cases, 10 showed a pathogenic germline mutation in the MMR genes (4 in *MLH1*, 4 in *MSH2* and 2 in *MSH6*). Of the remaining four cases, two showed hypermethylation of the *MLH1* gene promoter and two, V600E *BRAF* mutation. The other two patients showed lack of expression of MMR proteins but none of the above molecular alterations was detected; therefore, these cases could be defined as Lynch-like syndrome cases (Table 4). Regarding IOCRC, MSI was present in 4 of the 67 tumors that could be analyzed. Of these 4 tumors, MSI was due to MMR germline mutations in 3 cases (2 had a mutation in *MSH2*, and 1 in *MSH6*). The other one was a sporadic tumor with hypermethylation of the *MLH1* gene promoter. Finally, 9 tumors from LOCRC showed MSI. In this group, MSI was mostly due to *BRAF* mutations and/or hypermethylation of the *MLH1* gene promoter (eight cases). The other case carried a mutation in the *MSH2* gene.

According to *KRAS* mutations, there were no significant differences between groups, although there was some gradual increase (39%, 48% and 56%, regarding EOCRC, IOCRC and LOCRC subsets, respectively).

## Discussion

In the last decades, CRC has become a major concern worldwide given its high prevalence [1,2]. Although the incidence of this malignancy increases with age, a disturbing trend is being observed in the last years in which the prevalence of sporadic cases in young adults seems to be increasing [13]. As a consequence, several studies focused on improving the characterization of this disease according to the age of onset [13,17]. At present, the clinical manifestations of sporadic EOCRC are known to differ from those observed in LOCRC. Furthermore, molecular differences are also present, and some alterations occurring with different frequencies in both age groups have been reported [13,17]. However, some publications analyzing differences between early and late-onset CRC defined a specific cut-off age (usually 50 y/o), to divide them into both categories [18], while others compared both without taking into account intermediate ages in order to reach more accuracy in the comparisons [9,11]. In the present study, we aimed to determine what happens with patients with an intermediate age-of-onset of the disease, who do not belong to either EOCRC or LOCRC, and whether they could be considered a boundary group or a different group by itself, within CRC.

Firstly, we compared clinicopathological and familial features of a cohort of 272 patients differing in the age of onset (98 EOCRC, 83 IOCRC and 91 LOCRC). In our series, IOCRC bore more resemblance to EOCRC in terms of sex ratio, stage and survival, whereas it bore more resemblance to LOCRC in terms of colon location, development of polyps during follow-up, and a trend to developing S- and MCRC. Interestingly, IOCRC behaved as a different entity in histological terms, with mucin and signet ring cells being rare in this group (Table 1). Although some authors have related mucin production with a poor prognosis [19–21], in our series the mucinous component did not seem to be related to the prognosis of the disease; features such as overall survival and disease-free survival were very similar to those observed in EOCRC. The average number of polyps during follow-up was significantly higher in IOCRC in comparison with EOCRC and LOCRC. Finally, regarding family history of cancer, cases fulfilling the Amsterdam II criteria as well as families with aggregation for Lynch-related neoplasms were more common in EOCRC; the familial component decreased, and therefore the number of sporadic cases increased, in a progressive manner to IOCRC and hence to LOCRC (Table 1).

Regarding the type and duration of symptoms until diagnosis, LOCRC was the group with the lowest number of symptoms at the time of diagnosis (p = 0.018). In our series, IOCRC behaved as a transitional group between EOCRC and LOCRC. Accordingly, the symptoms and their duration in patients diagnosed at intermediate ages were not specific, but a gradation was observed from those most frequent in EOCRC to those most frequent in LOCRC (Table 2). Thus, abdominal symptoms and changes in bowel habit became less frequent as the age at diagnosis increased, although statistical significance was only reached in the first case (p = 0.005, Table 2). Incidental diagnosis was more frequent in LOCRC, although the causes responsible for these findings were different in each age group. While anemia was the main cause in elderly patients (possibly indicating a special awareness of looking for CRC underlying this situation), in the younger groups the screening programs became more important, especially in IOCRC. This fact was not surprising, considering that the recommendations for CRC screening in our environment include periodic examinations for all individuals without risk factors after the fifth decade of life, or at lower ages for individuals with a family history or other predisposing factors [22].

Apart from the age at diagnosis, we also compared the clinical manifestations depending on tumor location and stage of the disease (S1 and S2 Tables). Abdominal and unspecific symptoms and incidental diagnoses were more frequent in right-sided tumors. By contrast, intestinal bleeding and changes in bowel habit were most frequently observed in rectal tumors, whereas in left-sided tumors bowel obstruction was predominant. Regarding the correlation with tumor stages, we observed that advanced neoplasms usually showed a higher number of symptoms and a higher frequency of appearance of all symptoms studied, except for changes in bowel habit (p = 0.008). As expected, incidental diagnoses were more frequent in early stages as a result of the widespread implementation of population screening programs (p<0.001). To further asses the relationship between symptoms and tumor stages, we included the age at diagnosis as a variable in order to detect possible differences between the groups (Table 3). The trend of the results was along the same line as the one obtained without making a differentiation according to age. However, it is important to point out that IOCRC was the group in which the clinical manifestations differed most depending on the stage of the disease at the moment of diagnosis. Our results confirm one of the main findings of the study of Chen et al. [18] on age-of-onset and symptoms and their duration to diagnosis, even though they analyzed EOCRC, as we did, but compared it with the rest of the population (older than 50) as a whole. EOCRC cases experienced significantly longer symptom duration, as we found, showing in our case the same results for IOCRC cases. However, in contrast to their finding that EOCRC cases with stage III or IV disease had shorter symptom and work-up periods than those with stage I or II disease, we found a correlative progression between symptoms duration and advancing locoregional stages (I to III), but, in agreement with Chen et al. [18], it was shorter only in stage IV tumors. Whereas in our opinion our findings show a more likely influence of the delay of diagnosis within locoregional stages, and of biological tumor factors in relation with IV-stages, our data do underscore the necessity of a greater awareness of not only EOCRC, but also of IOCRC.

Lastly, we evaluated the tumors from a MSI point of view and observed that IOCRC showed the lowest rate of MSI, mainly due to germline mutations in the MMR genes, as well as a low rate of *BRAF* mutations (Table 4). These findings seem to confirm our premise that the IOCRC group may be a transitional group between EOCRC and LOCRC, because although there was a decrease in cases with a family history of CRC from EOCRC to the group with more sporadic tumors (LOCRC), the rate of LS cases decreased at the same time, and the number of MSI cases within LOCRC is mainly due to sporadic cases. According to *KRAS* mutations, there was some gradual increase (39%, 48% and 56%, regarding EOCRC, IOCRC and LOCRC subsets, respectively), being slightly like other publications [23].

From a clinical point of view, IOCRC appears to behave as a transitional group between EOCRC and LOCRC, with common features with both groups, but also with group-specific features. The variation of the familial component throughout the three categories and its differential MSI component (Lynch syndrome component and sporadic cases) is interesting. In order to find possible differential molecular bases for CRC according to the age-of-onset, future analyses should be carried out maintaining an upper limit of 50 y/o for EOCRC, and comparing this group with both the IOCRC and LOCRC categories, or by using exclusively an LOCRC group, but defined with a much higher age of onset than 50 y/o (e.g. 70 y/o), unless large demographic studies could define a possible age-of-onset cut-off between only two main different age-of-onset CRC populations. Finally, concerning symptoms and their duration until diagnosis, and excluding cases with familial cancer history, the awareness for EOCRC diagnosis should be extended to IOCRC.

## Supporting information

S1 Table Clinical manifestations according to tumor location. (DOC)

S2 Table Correlation between type/duration of the symptoms and tumor stage at the time of diagnosis. (DOCX)
