# Supplementary material for: Intermediate-onset colorectal cancer: A clinical and familial boundary between both early and late-onset colorectal cancer
Source: PLoS One. 2019 May 16;14(5):e0216472. doi: 10.1371/journal.pone.0216472 (PMC6521992; doi:10.1371/journal.pone.0216472)
Supplement: S1 Table — (DOC) [file pone.0216472.s001.doc]

S1 Table. Clinical manifestations according to tumor location.

|  | Right colon | Left colon | Rectum | p-value^1^ |
| --- | --- | --- | --- | --- |
| No. of patients | 71 | 77 | 83 | - |
| Intestinal bleeding | 13 (18) | 25 (32) | 48 (58) | <0.001 |
| Abdominal symptoms | 23 (32) | 19 (25) | 9 (11) | 0.005 |
| Constitutional syndrome | 14 (20) | 16 (21) | 22 (27) | NS |
| Unspecific symptoms | 12 (17) | 7 (9) | 4 (5) | 0.042 |
| Incidental diagnosis: | 24 (34) | 22 (29) | 12 (14) | 0.015 |
| Changes in bowel habit | 13 (18) | 14 (18) | 28 (34) | 0.030 |
| Bowel obstruction | 4 (6) | 9 (12) | 4 (5) | NS |

Data shown in parenthesis represent percentages. ^1^Statistical comparison was performed using Pearson’s Chi Square (χ^2^) test. No.: Number. NS: Not significant.
