# Supplementary material for: Intermediate-onset colorectal cancer: A clinical and familial boundary between both early and late-onset colorectal cancer
Source: PLoS One. 2019 May 16;14(5):e0216472. doi: 10.1371/journal.pone.0216472 (PMC6521992; doi:10.1371/journal.pone.0216472)
Supplement: S2 Table — (DOCX) [file pone.0216472.s002.docx]

S2 Table. Correlation between type/duration of the symptoms and tumor stage at the time of diagnosis.

|  | n | I | II | III | IV | p-value^1^ |
| --- | --- | --- | --- | --- | --- | --- |
| No. of patients | 231 | 49 (21) | 78 (34) | 46 (20) | 58 (25) | - |
| Intestinal bleeding:  Associated  Isolated  Self-limited | 86  45  16  25 | 14 (16)  3 (7)  2 (13)  9 (36) | 28 (33)  17 (37)  4 (25)  7 (28) | 23 (27)  13 (29)  6 (37)  4 (16) | 21 (24)  12 (27)  4 (25)  5 (20) | NS |
| Abdominal symptoms | 51 | 4 (8) | 15 (29) | 11 (22) | 21 (41) | 0.005 |
| Constitutional syndrome | 52 | 3 (6) | 18 (35) | 11 (21) | 20 (38) | 0.006 |
| Anorectal (excluding bleeding) | 15 | 1 (7) | 8 (53) | 3 (20) | 3 (20) | NS |
| Unspecific symptoms | 23 | 2 (9) | 6 (26) | 5 (22) | 10 (43) | NS |
| Incidental diagnosis:  Anemia  Screening  Others | 58  24  25  9 | 28 (48)  5 (21)  18 (72)  5 (56) | 19 (33)  14 (58)  4 (16)  1 (11) | 5 (9)  4 (17)  1 (4)  0 (0) | 6 (10)  1 (4)  2 (8)  3 (33) | <0.001 |
| Changes in bowel habit | 55 | 3 (5) | 23 (43) | 15 (27) | 14 (25) | 0.008 |
| Duration of symptoms (months) | 4.11 ± 6.73 | 1.44 ± 3.99 | 4.15 ± 7.03 | 7.02 ± 9.55 | 3.88 ± 4.08 | <0.001^2^ |

Data shown in parenthesis represent percentages. 1Statistical comparison was performed using Pearson’s Chi-Square (χ2) test. ^2^Statistical comparison was performed using the Kruskal-Wallis test. No.: Number. NS: Not significant.
